# Counter-clockwise vortical blood flow in the main pulmonary artery in a patient with patent ductus arteriosus with pulmonary arterial hypertension: a cardiac magnetic resonance imaging case report

**DOI:** 10.1186/s12880-016-0150-z

**Published:** 2016-08-08

**Authors:** Gert Reiter, Ursula Reiter, Gabor Kovacs, Gabriel Adelsmayr, Andreas Greiser, Aurelien F. Stalder, Horst Olschewski, Michael Fuchsjäger

**Affiliations:** 1 Siemens Healthcare, Graz, Austria; 2 Division of General Radiology, Department of Radiology, Medical University of Graz, Auenbruggerplatz 9/P, A-8036 Graz, Austria; 3 Division of Pulmology, Department of Internal Medicine, Medical University of Graz, & LBI for Lung Vascular Research Graz, Graz, Austria; 4 Siemens Healthcare, Erlangen, Germany

**Keywords:** Pulmonary arterial hypertension, 4D blood flow, Patent ductus arteriosus, Cardiac magnetic resonance imaging

## Abstract

**Background:**

In patients with pulmonary hypertension (PH), duration of vortical blood flow along the main pulmonary artery enables estimation of the mean pulmonary arterial pressure (mPAP) non-invasively. It remains to date not known, if this method is applicable in patients with pulmonary arterial hypertension (PAH) and abnormal aortic-to-pulmonary shunting.

**Case presentation:**

The present case analyzes the effect of a patent ductus arteriosus (PDA) on pulmonary artery flow patterns in PAH (mPAP from right heart catheterization, 75 mmHg). PH-associated vortical blood flow, which is typically observed rotating in a clockwise direction when viewed in right ventricular outflow tract orientation, was found nested in PDA left-to-right shunting. Even though rotating counter-clockwise, duration of vortical flow translated into correct non-invasive mPAP estimate.

**Conclusions:**

This case indicates that PH-associated vortex rotation is not restricted to clockwise direction, and that vortex-based estimation of elevated mPAP might also be feasible in patients with PAH and PDA.

**Electronic supplementary material:**

The online version of this article (doi:10.1186/s12880-016-0150-z) contains supplementary material, which is available to authorized users.

## Background

In patients with pulmonary hypertension (PH) without abnormal aortic-to-pulmonary shunting, PH is associated with vortical blood flow along the main pulmonary artery, whose duration is linearly related to elevated mean pulmonary arterial pressure (mPAP) [1, 2]. Typically, vortex formation takes place at the posterior wall of the main pulmonary artery, and the vortex rotates in a clockwise direction when viewed in the right ventricular outflow tract (RVOT) orientation [1, 3–5].

Pulmonary arterial hypertension (PAH) is a complication in patients with patent ductus arteriosus (PDA), and the retrograde blood flow in the main pulmonary artery caused by left-to-right shunting via the PDA might modify flow patterns typically observed in PH [6]. Potentially, it might affect the presence of a vortex per se, the vortex location, the rotational direction, and also the relationship between vortex duration and mPAP. To approach these issues, pulmonary arterial blood flow patterns were analyzed in a patient with PDA and PAH.

## Case presentation

A 21-year-old male patient (Table 1) with PDA and PAH (mPAP, 75 mmHg; systolic/diastolic pulmonary arterial pressures, 102/58 mmHg; systolic/diastolic systemic arterial pressures, 112/67 mmHg; pulmonary arterial wedge pressure, 9 mmHg; right atrial pressure, 7 mmHg; cardiac index, 3.4 L/min/m^2^, pulmonary vascular resistance, 11.5 Wood units) was referred for 3 T cardiac magnetic resonance (MR) function and 4D flow imaging (Magnetom Trio, Siemens Healthcare, Erlangen, Germany) after right heart catheterization (RHC) within a prospective study approved by the local ethical review board (ClinicalTrials.gov Identifier NCT01725763). Cardiac function was assessed by cine steady-state free precession real-time imaging during free breathing. Volumetric evaluation revealed enlarged left (LV) and right (RV) ventricular volumes (end-diastolic volume, LV: 126 mL/m^2^, RV: 121 mL/m^2^; end-systolic volume, LV: 55 mL/m^2^, RV: 70 mL/m^2^; ejection fraction, LV: 56 %, RV: 42 %), normal left (65 g/m^2^) and increased right ventricular mass (61 g/m^2^). Left (LA) and right (RA) atrial volumes (maximum volume, LA: 39 mL/m^2^, RA: 65 mL/m^2^; minimum volume, LA: 19 mL/m^2^, RA: 30 mL/m^2^) were within normal ranges [7].

**Table 1:** Timeline

1993:	Preterm birth (35^th^ week of pregnancy); bronchopulmonary dysplasia; severe obstructive pulmonary disease
1994:	Echocardiography: enlarged RV and RA; normal LV and LA; small PDA with left-to-right shunt
2006:	Aortic angiography: PDA diameter, 3.5 mm
	Right heart catheterization and PDA trial occlusion: mPAP, 70 mmHg; RV pressures did not significantly decrease after trial occlusion
	Echocardiography: RV/LV/PV diameter, 24/55/34 mm; maximal pressure gradient across PDA, 16 mmHg
2006 – 2014:	Bosentan therapy and close meshed therapy monitoring
2008:	Echocardiography: RV/LV/PV diameter, 28/54/35 mm; maximal pressure gradient across PDA, 16 mmHg
	Right heart catheterization and PDA trial occlusion: PAP, 94/53/70 mmHg; RAP, 4 mmHg. RV pressures did not significantly decrease after trial occlusion
2013:	Echocardiography: RV/LV/PV diameter, 24/59/39 mm
2014:	Right heart catheterization: see case presentation. Clinical classification: PAH associated with congenital heart disease (PDA) according to positive treatment effect of Bosentan
	Cardiac MR: see case presentation
since 2014:	Macitentan therapy

*PDA*, patent ductus arteriosus, *RV* right ventricle, *LV* left ventricle, *PV* pulmonary valve, *RVP* systolic/diastolic/mean, right ventricular pressure, *PAP* systolic/diastolic/mean, pulmonary arterial pressure, *RAP* right atrial pressure, *PAH* pulmonary arterial hypertension

MR 4D flow data were acquired in free breathing, covering the heart, pulmonary artery and aorta with gapless slices of a retrospectively ECG-gated, segmented, two-dimensional spoiled gradient-echo-based cine phase-contrast sequence with three-directional velocity encoding in RVOT orientation. Velocity fields were calculated, visualized and analyzed by dedicated prototype software (4D Flow V2.4, Siemens Healthcare, Erlangen, Germany) [8].

**Fig. 1:**
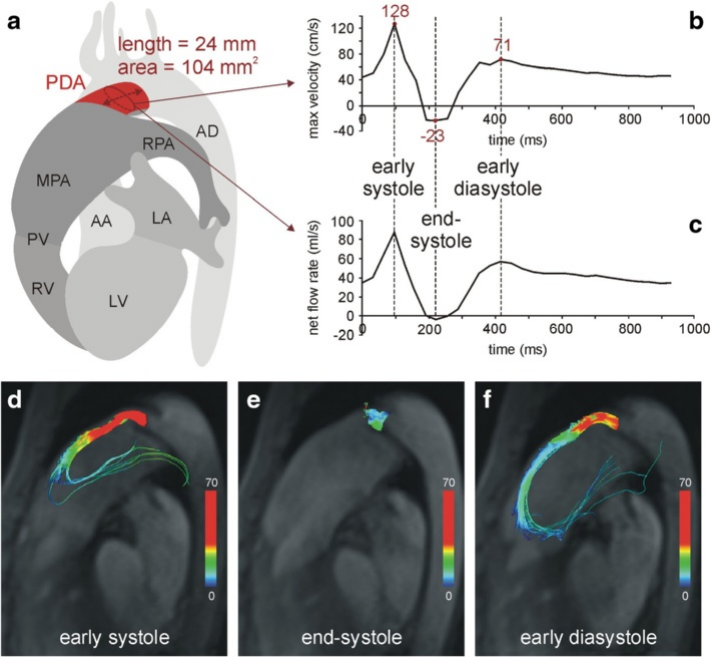
4D flow evaluation of the PDA. Schematic 3D anatomy (**a**) of the PDA and surrounding cardiovascular structures based on 3D reconstruction of the anatomical phase contrast images. PDA length and time-averaged cross-sectional area at the center of the PDA were evaluated by multi-planar reformation. Time courses of maximal velocity (**b**) and net flow rate (**c**) across the central cross-section of the PDA demonstrate an early systolic and an early diastolic left-to-right peak and small right-to-left flow at end-systole. Velocity-color-encoded streamlines originating from PDA at early systole (**d**), end-systole (**e**) and early diastole (**f**) projected onto multi-planar reformatted anatomical images reflect these bi-phasic PDA flow characteristics. In early systole (**d**) and diastole (**f**) there is fast left-to-right flow through the PDA that reverses direction in the main pulmonary artery. In end-systole (**e**) blood spirals from right-to-left but no streamlines (and particles in Additional file 1: in the online-only Data Supplement) enter the aorta. PDA = patent ductus arteriosus; MPA = main pulmonary artery; RPA = right pulmonary artery; PV = pulmonary valve; AD = aorta descendens; AA = aorta ascendens, LV = left ventricle, RV = right ventricle, LA = left atrium

Multi-planar reformatted anatomical phase contrast images demonstrated a dilatated main pulmonary artery (cross-sectional area, 15.8 cm^2^), and a tubular ductus arteriosus 24 mm in length and 104 mm^2^ in cross-sectional area connecting the proximal descending aorta to the left pulmonary artery near its origin (Fig. 1). Time-course of PDA maximal velocity (Fig. 1b) and net blood flow rate (Fig. 1c) during the cardiac cycle, as well as streamlines (Fig. 1d, e, f) and particle traces (see Additional file 1) originating from the PDA, demonstrated biphasic left-to-right shunting without Eisenmenger’s syndrome. The systolic/diastolic pressure gradients between aorta and pulmonary artery of 7/2 mmHg, calculated from systolic/diastolic peak velocities across the PDA (Fig. 1b) via the Bernoulli equation (pressure gradient in mmHg = 4 · v^2^, with v being the PDA peak velocity in m/s), matched the difference between systemic and pulmonary systolic/diastolic pressures. Shunt volume across the PDA was 33 mL, which was in agreement with the difference of measured net forward volumes in the ascending aorta (120 mL) and main pulmonary artery (81 mL). The resulting pulmonary-to-systemic flow ratio was 0.7.

**Fig. 2:**
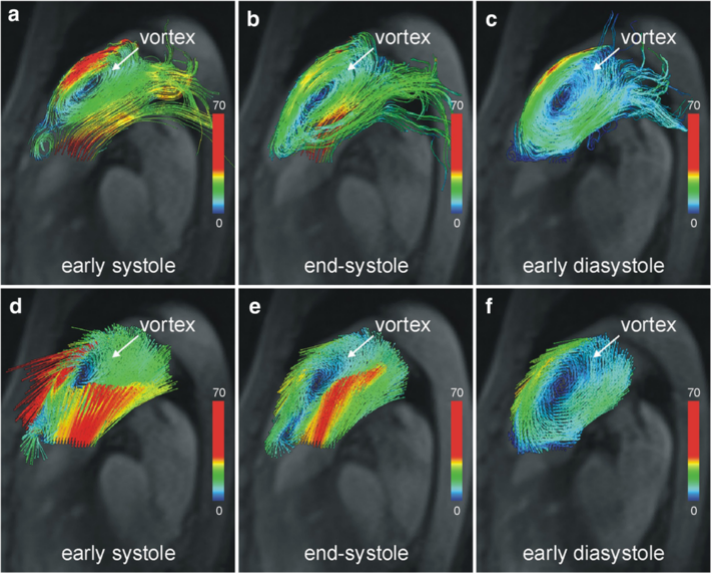
Vortical blood flow in the main pulmonary artery. Velocity-color-encoded streamlines (**a**, **b**, **c**) and 3D velocity vectors (**d**, **e**, **f**) projected onto multi-planar reformatted anatomical images demonstrate counter-clockwise rotating vortical blood flow nested in bi-directional pulmonary flow caused by PDA. This structure is present throughout the entire cardiac cycle (Additional file 2: in the online-only Data Supplement)

Streamlines (Figs. 2a, b, c) and particle traces (see Additional file 2) originating from the main pulmonary artery, as well as 3D velocity vector fields (Figs. 2d, e, f), demonstrated that left-to-right ductal shunting caused retrograde blood flow along the anterior wall of the main pulmonary artery, reversing direction from retrograde to anterograde above the pulmonary valve. This anterograde flow adhered to the forward flow through the pulmonary valve during systole.

**Fig. 3:**
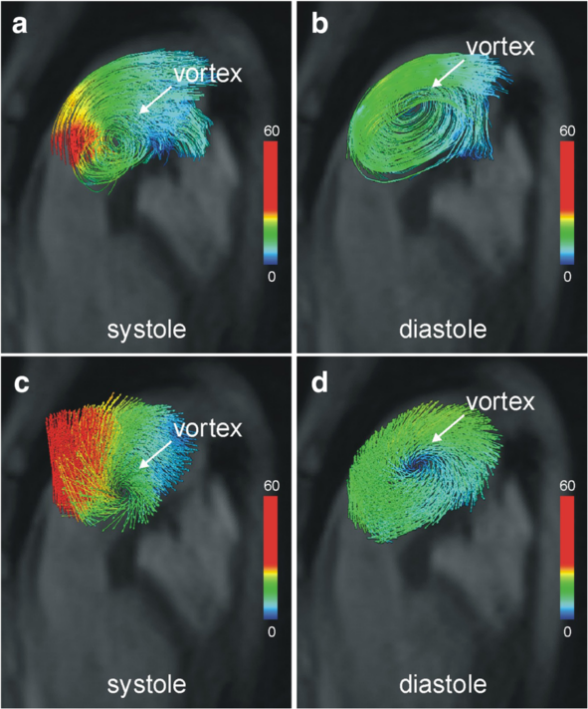
Typical vortical blood flow in the main pulmonary artery in a patient with pulmonary arterial hypertension (mPAP = 82 mmHg) without PDA. Velocity-color-encoded streamlines (**a**, **b**) and 3D velocity vectors (**c**, **d**) projected onto multi-planar reformatted anatomical images demonstrate clockwise rotating vortical blood flow and forward flow along the posterior wall of the main pulmonary artery

The typical flow pattern in patients with PAH (Fig. 3) - i.e., vortex formation at the posterior wall of the main pulmonary artery - was not seen. Instead there was a vortex at the anterior wall of the main pulmonary artery nested between retrograde and anterograde PDA-caused blood flow and rotating counter-clockwise (i.e., opposite the typical clockwise direction) (Fig. 2). The duration of this vortical blood flow was the complete cardiac interval (t_vortex_ = 100 %), which translated into mPAP of 79 mmHg (according to the formula mPAP in mmHg = 16 + 0.63 · t_vortex_) [2] and was very close to the value measured during RHC.

## Discussion

Irrespectively of the underlying etiology of PH, vortical blood flow along the main pulmonary artery has been identified as a marker of elevated mPAP [1–3]. The formation of this vortical flow has been attributed to the appearance of a thickened boundary layer in the main pulmonary artery caused by increased pulmonary vascular resistance, reduced compliance and altered pulse wave reflection [1, 3]. Owing to the curvature of the vessel this thickened boundary layer is located at the posterior wall of the main pulmonary, which leads to the formation of a clockwise rotating vortex.

In the present case of PDA and PAH, vortex formation is observed at the anterior wall of the main pulmonary artery and its rotational direction is counter-clockwise. This flow topology has not been described neither in PH in general, nor in PAH in particular [1–5, 9]. It could be speculated that the PDA jet which is pressing the pulmonary arterial forward flow against the posterior vessel wall impedes vortex formation in this region. Jet stream boundaries on the other hand give generally rise to zones of turbulent mixing [10]; PDA jet could therefore favor vortex formation at the anterior wall of the main pulmonary artery.

In PH patients without PDA, duration of vortical blood flow in the main pulmonary artery was found to be closely related to mPAP [2]. Notably, mPAP calculated from the duration of vortical blood flow in the present case revealed good agreement with the mPAP measured by RHC. It could therefore be conjectured that the observed counter-clockwise rotating vortex is similarly related to inadequate release of blood from the main pulmonary artery into the pulmonary vasculature as the clockwise rotating vortex in patients with elevated mPAP without PDA. This would mean that the vortex – irrespectively of its rotational direction – represents a mechanism of “kinetic energy storage”, counteracting the increased vascular resistance and the reduced compliance in PH. Clinical studies including patients with PDA with/without PAH and with/without Eisenmenger’s syndrome are needed to gain better insights into the complex hemodynamics of PDA with PAH.

## Conclusions

The current case indicates that vortex-based estimation of elevated mPAP might be feasible in patients with PDA. However, this case also shows that in a patient with PDA, the location and direction of vortical blood flow may differ from those typically seen in patients with PH without PDA.

## Abbreviations

mPAP, mean pulmonary arterial pressure; MR, magnetic resonance; PAH, pulmonary arterial hypertension; PDA, patent ductus arteriosus; PH, pulmonary hypertension; RHC, right heart catheterization; RVOT, right ventricular outflow tract; t_vortex_, period of existence of vortical blood flow

## Additional files

Additional file 1: Velocity-color-encoded particle trace visualization of blood flow with particles seeded in the PDA. (MP4 428 kb) Additional file 2: Velocity-color-encoded particle trace visualization of blood flow with particles seeded uniformly in the main pulmonary artery. (MP4 1415 kb)
